# Electroencephalographic features of discontinuous activity in anesthetized infants and children

**DOI:** 10.1371/journal.pone.0223324

**Published:** 2019-10-03

**Authors:** Uday Agrawal, Charles B. Berde, Laura Cornelissen

**Affiliations:** Department of Anesthesiology, Critical Care and Pain Medicine, Boston Children’s Hospital, Harvard Medical School, Boston, Massachusetts, United States of America; Massachusetts General Hospital, UNITED STATES

## Abstract

**Background:**

Discontinuous electroencephalographic activity in children is thought to reflect brain inactivation. Discontinuity has been observed in states of pathology, where it is predictive of adverse neurological outcome, as well as under general anesthesia. Though in preterm-infants discontinuity reflects normal brain development, less is known regarding its role in term children, particularly in the setting of general anesthesia. Here, we conduct a post-hoc exploratory analysis to investigate the spectral features of discontinuous activity in children under general anesthesia.

**Methods:**

We previously recorded electroencephalography in children less than forty months of age under general anesthesia (n = 65). We characterized the relationship between age, anesthetic depth, and discontinuous activity, and used multitaper spectral methods to compare the power spectra of subjects with (n = 35) and without (n = 30) discontinuous activity. In the subjects with discontinuous activity, we examined the amplitude and power spectra associated with the discontinuities and analyzed how these variables varied with age.

**Results:**

Cumulative time of discontinuity was associated with increased anesthetic depth and younger age. In particular, age-matched children with discontinuity received higher doses of propofol during induction as compared with children without discontinuity. In the tens of seconds preceding the onset of discontinuous activity, there was a decrease in high-frequency power in children four months and older that could be visually observed with spectrograms. During discontinuous activity, there were distinctive patterns of amplitude, spectral edge, and power in canonical frequency bands that varied with age. Notably, there was a decline in spectral edge in the seconds immediately following each discontinuity.

**Conclusion:**

Discontinuous activity in children reflects a state of a younger or more deeply anesthetized brain, and characteristic features of discontinuous activity evolve with age and may reflect neurodevelopment.

## Introduction

Discontinuous electroencephalographic (EEG) activity consists of alternating periods of continuous activity and periods of suppression. In the neonatal literature, suppressions related to discontinuity are defined as electrical activity less than 25 μV in a majority of the EEG leads that persists for 2 seconds or more [1]. If the suppression is less than 5 μV and non-reactive, then it is termed burst suppression [2], though alternative definitions have also been proposed [3,4]. This discontinuous pattern of activity is thought to reflect ongoing brain development in premature healthy children [2]. In adults and full-term children, discontinuous activity reflects a profoundly inactivated brain, often reflects pathological states such as encephalopathy [1,5], or a stressed brain following cardiac bypass surgery [6] and is predictive of adverse neurological outcomes [7]. Discontinuous activity has also been observed in children under general anesthesia [8–12]. We recently reported that more discontinuity events occur in younger children and at a higher anesthetic depth [11].

Discontinuity or burst suppression, likely represent weak connectivity, possibly involving interruption of thalamocortical and cortico-cortical dynamics. To understand the neurophysiology of anesthesia in the infant brain and the dynamics of discontinuity, we can evaluate EEG data which contains several structured patterns that relate to brain state. In adults, for example, increased alpha (8-12Hz) oscillations appear during loss of consciousness and are sustained during maintenance anesthesia, reflecting thalamocortical and corticocortical connectivity [13]. In contrast, alpha oscillations in infants are absent from birth until 3-months, gradually appearing until they reflect an adult-like pattern at around 10-months old [14,15].

Interest in EEG signatures of brain state, including suppression has increased because EEG is now more widely used in the operating room and intensive care unit to monitor brain state for adult care. Brain monitoring algorithms that automatically detect anesthetic depth, bursting and/or burst suppression perform poorly in young children due to a lack of translation between adult-derived algorithms [16].

Despite the presence of discontinuity in children under anesthesia, spectral and temporal EEG properties are poorly described. Here, we conduct a post-hoc exploratory analysis in previously collected data containing discontinuous activity in infants aged 0 to 40 months old under general anesthesia. Segmentation of the EEG into discontinuity-events and inter-discontinuity events (preceding and immediately following) is an essential first stage for more advanced analysis. Outcome measures include time-frequency properties and signal amplitude. We hypothesize that by characterizing the intensity and structure of the oscillations around discontinuity, we can improve our understanding of the brain state in infants and children during general anesthesia.

## Materials and methods

### Study design

This is a retrospective study with secondary analysis of previously published data describing incidence of discontinuous EEG activity in children during sevoflurane general anesthesia [11]. In this complementary paper, we focus on characterizing the EEG spectral and temporal properties.

### Ethics approval

This study was approved by the Boston Children’s Hospital Institutional Review Board and classified as ‘no more than minimal risk’. Informed written consent was provided by the parents/caregivers.

### Patient selection

Children were recruited from Boston Children’s Hospital (BCH) preoperative clinic from December 2012 to August 2017. Eligibility criteria included ages 0 to 40 months-old, American Society of Anesthesiologists’ physical status I–II, and requiring surgery below the neck. Exclusion criteria were (i) congenital malformations or other genetic conditions thought to influence brain development, (ii) neurological or cardiovascular disorder, or (iii) born <32 weeks postmenstrual age.

One subject previously reported on in [11] was excluded due to technical artifact, and one additional subject was recruited and included in this analysis.

### Anesthetic management

Anesthetic management and intraoperative events were recorded from the internal Anesthesia Information Management System. End tidal sevoflurane (etSevo) was collected from the anesthetic monitoring system in real time with a sampling frequency of 1 Hz using ixTrend (ixcellence, Wildau, Germany). Timing of propofol bolus administration was confirmed by manual inspection of video recordings time locked to the EEG (Xltek EMU40; Natus Medical Inc., Oakville, Ontario, Canada). Videos were reviewed frame-by-frame to identify the time point (seconds) for propofol bolus administration. Clinical information and demographics were collected from electronic medical records.

### EEG recording

Whole-head EEG data were recorded using an Xltek recording system (EMU40EX Natus Medical Inc., Ontario Canada). The EEG data were recorded from 0 to 500Hz, sampling rate of 1024Hz, a 16-bit resolution and 300nV quantization. An EEG cap (WaveGuard; Advanced NeuroTechnology, Enschede, the Netherlands) was placed on patients before anesthesia administration started. A minimum of 33 electrodes were positioned according to the modified international 10/20 electrode placement system. Reference and ground electrodes were located at Fz and AFz, respectively. Impedance was minimized by massaging the skin with an EEG prepping gel (Nu-Prep gel; D.O. Weaver & Co., Aurora CO, USA), and electrode contact was optimized using a conductive gel (Onestep-Clear gel; H+H Medical Devices, Dulmen, Germany).

### EEG analysis

The EEG data was bandpass filtered from .1–100 Hz and down sampled to 256 Hz prior to analysis. Two authors (U.A., L.C.) visually inspected all EEG data for each subject and manually selected data segments free of noise and artifacts for analysis.

### Epoch identification

We manually identified EEG data segments with discontinuous activity (+/- burst suppression). A discontinuous event was defined as a period of EEG activity below 25 μV lasting for 2 seconds or greater and observed across the majority of electrodes. To obtain precise onset and offset times of individual discontinuity events, a variance-based threshold method was performed (modified from *Lewis et al*. [17]). For epoch identification, the EEG signal was high-pass filtered at 3 Hz, and the variance of the filtered signal was computed in 125 millisecond sliding windows. The threshold was defined as the mean variance plus four standard deviations from the mean variance during a manually identified discontinuity event. “Onset of Discontinuity” was defined as the first variance crossing below threshold. “Offset of Discontinuity” was defined as the subsequent variance crossing greater than threshold.

For EEG segments of interest, we computed the EEG properties for the electrode F7. We analyzed only one electrode because discontinuity events occurred simultaneously across all leads (to the limit of our temporal resolution). We focused on F7 because we have previously observed notable changes in frontal oscillations related to anesthetic depth and development [14,15].

### Time-frequency analysis

For EEG segments of interest, we computed the power spectrum and spectrogram, and spectral edge. Power spectra and spectrograms were generated using multitaper methods as implemented in the Chronux toolbox (http://chronux.org/) [18]. The power spectral density quantifies the frequency distribution of power within an EEG electrode signal. The spectrogram is a time-varying version of the power-spectral density, estimated on consecutive windows of EEG data. In these spectrograms, frequencies are arranged along the y-axis and time along the x-axis, and power is indicated by color on a decibel scale. Parameters for the power spectra analysis were: time bandwidth product TW = 3 and number of tapers K = 5. Parameters for spectrograms were: time bandwidth product TW = 2, number of tapers K = 3, window length T = 2 seconds with a 100-millisecond overlap. Spectral edge, which is the frequency below which 95% of the power is located, was also computed.

### EEG segments of interest

#### (1) Pre-discontinuity event

To characterize the temporal properties in spectral activity before a discontinuity event, we computed the power spectra and spectral edge of two 10 second segments–(i) a five to one-minute interval (or -300s to -60s) preceding the first discontinuity, and (ii) a one-minute interval (or -60 to 0s) preceding the first discontinuity.

#### (2) Post-discontinuity event

We computed the spectral properties in the first 2s of each artifact-free post-discontinuity interval for all discontinuous events (n = 186 events). We also computed the spectral edge during a 1s segment of discontinuity preceding each post-discontinuity interval.

We then investigated how spectral characteristics change over the course of a post-discontinuity interval. We grouped each of the artifact-free post discontinuity intervals (n = 186 events) into the following age groups: 0–3 months (n = 98), 4–6 months (n = 37), 7–9 months (n = 38), and 9 months and older (n = 23). We then separately compared the spectral edge for the first 1.5s of the post-discontinuity interval and the subsequent 1.5s of the post-discontinuity interval.

### Time-amplitude analysis

We examined how the amplitude of the discontinuities, or suppressions, and the amplitude of the activity immediately following the discontinuities, which we refer to as post-discontinuity intervals, changed with age. The root mean squared amplitude during 1s of discontinuity (n = 186 events) across subjects was computed. The root mean squared amplitude during the immediate 2s following each of the discontinuities was computed.

### Comparison of discontinuous activity with maintenance (moderate-levels) of anesthesia

To check whether any inherent differences were present in the EEG data of subjects with and without discontinuity, we compared the power spectra and spectral edges during a 60s segment of maintenance anesthesia in subjects who did- and did-not exhibit discontinuity events. Subjects were paired for age and when the difference between the etSevo was less than 1% (n = 20 subjects).

We also assessed whether the spectral characteristics of the post-discontinuity interval were similar to those observed during lighter stages of anesthesia. Power spectra of the two seconds following each discontinuity were compared with two seconds of stable activity during maintenance in the age groups 0–3 months (n = 98 events) and 4 months and older (n = 92 events).

### Statistical analysis

All analyses performed were post-hoc exploratory analyses from previously collected data [11].

**Fig 1:**
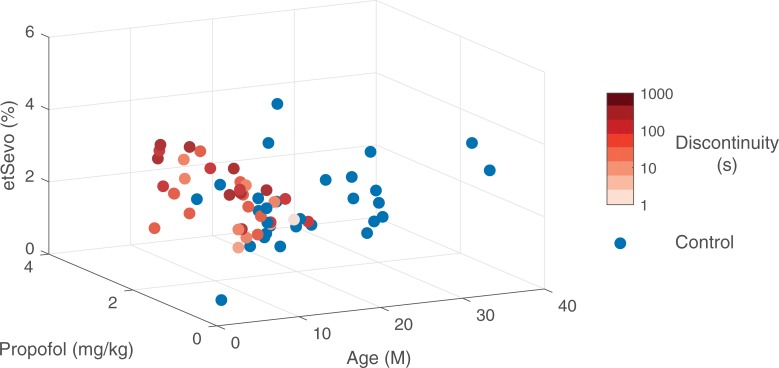
Postnatal age is negatively associated with and anesthetic depth positively associated with discontinuity. Age, median end tidal sevoflurane (etSevo) during induction, and cumulative propofol dose during induction in all subjects (n = 65). A multiple linear regression showed that younger age, increased sevoflurane, and increased propofol were associated with longer cumulative discontinuity during induction (F(4,61) = 6.02, p < .005) with an adjusted R^2^ of 0.19. Subjects with discontinuity (n = 35) in red (intensity ranges from 1 to 1000 seconds on a log scale) and without discontinuity in blue (n = 30).

Multiple linear regression was performed across all subjects to predict total time in discontinuity during induction with the following covariates: age, median etSevo concentration during induction, and cumulative dose of propofol boluses delivered during induction (n = 65), and the fit assessed using an F-test (i.e. Fig 1).

Linear mixed effects models were used to characterize the association between (1) amplitude and spectral features of the post-discontinuity intervals with age, while accounting for multiple observations per subject; and (2) to describe the association of age with i) spectral edge during discontinuity, ii) spectral edge during the post-discontinuity interval, iii) mean slow power, iv) mean delta power, and v) mean alpha power.

Comparison of the etSEVO and propofol dosing in subjects with and without discontinuity, and spectral edge was performed using the paired non-parametric Wilcoxon signed rank test.

Likelihood ratio tests were used to guide selection of the polynomial model, and confidence intervals for the regression curves were computed using bootstrap orders, and an order of two (i.e. a quadradic model) was chosen for each model [19]. Briefly, bootstrap samples were simulated by adding a normally distributed error to the collected data with a variance equal to the residual variance of the original regression. A new regression was performed on the bootstrap sample, and this process was repeated for 2000 simulated samples to generate the 95% confidence intervals. The differences between discontinuity and post-discontinuity interval amplitude and spectral edge were assessed by generating confidence intervals around the difference between the regression curves. Specifically, the difference between randomly drawn samples of the regression curves of discontinuity and the post-discontinuity interval were calculated to generate a 95% confidence interval, and the differences were considered statistically significant if the estimated confidence intervals did not include 0.

Confidence intervals for power spectra were generated using a frequency domain bootstrapping algorithm as described previously [14,20]. In brief, bootstrap samples (n = 2000) were drawn from the power spectra of the post-discontinuity interval and activity during maintenance and the median difference was computed at each frequency. The power at two frequencies was considered significantly different if a median difference of 0 was not in the 95% confidence interval.

### Source data

Data used to generate the figures are given in the Supporting Information (S1 File).

### Use of previously published data sets

The EEG data collected during general anesthesia have been reported elsewhere: specifically, as a descriptive study on discontinuity during deep levels of anesthesia at 0–3 years old [11], on spectral and coherence patterns during maintenance and emergence anesthesia at 0–6 months and 0–3 yrs old [14,15,21], and in evaluating machine-learning algorithms to predict anesthetic concentration in neonatal animals and human infants at 0–6 months old [22]. A statement on the use of previously published data sets is given in the Supporting Information (S2 File).

## Results

We retrospectively reviewed EEG recordings in children undergoing general anesthesia aged 0 to 40 months. First, we analyzed the predisposing factors to discontinuous activity, focusing on age and anesthetic depth. Second, we examined whether any notable patterns were present in the spectrograms of EEG preceding the onset of discontinuous activity. Third, we characterized spectral features of the discontinuous activity, focusing on the few seconds following each discontinuity event.

### Age and anesthetic depth associated with discontinuous activity

In subjects who exhibited discontinuity, the median age was 6 months-old [interquartile range (IQR): 3.1 to 7 months-old], and in those who did not, the median age was 10.7 months-old (IQR: 6.1 to 18.3 months-old). We characterized the relationship between age, anesthetic depth, and discontinuous activity in children undergoing anesthesia in order to understand the factors which may contribute to the onset of the brain state. In particular, we separately analyzed the effects of etSevo, which was the primary anesthetic used for the operations we studied, and propofol, which was often delivered as a bolus to rapidly increase anesthetic depth during induction.

In subjects who exhibited discontinuity, the median etSEVO was 2.8% (IQR: 2.5 to 3.5%). In subjects who were administered a propofol bolus and then exhibited a discontinuity, the median propofol bolus was 2.1 mg/kg (IQR: 1.6 to 2.4mg/kg). The median cumulative duration of discontinuity during induction was 138s (IQR: 38.5 to 324s). We performed a multiple linear regression with age, etSevo, and propofol to predict time in discontinuity and found a significant regression equation (F(4,61) = 6.02, p < .005) with an adjusted R^2^ of 0.19 (Fig 1). Both etSevo and propofol dose were positively associated with length of discontinuous activity (etSevo: coefficient 80.83, p < 0.017, propofol: coefficient 62.39, p < 0.0099), and age was negatively associated with length of discontinuous activity (coefficient -8.62, p < .0069).

**Fig 2:**
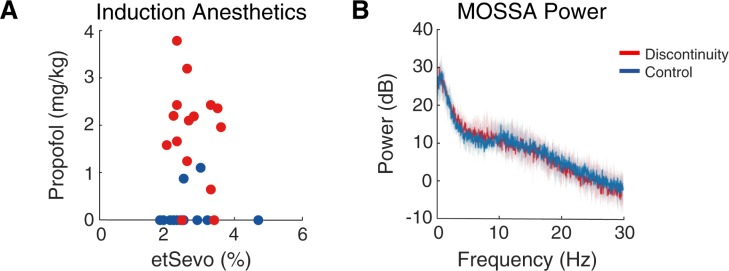
Propofol boluses are associated with increased likelihood of discontinuity, but power spectra similar during maintenance between groups. (A) Age-matched subjects (n = 30) with discontinuity (red) received more propofol during induction than subjects without discontinuity (blue) (Wilcoxon Sign-Rank, p < .0005). (B) Age-matched subjects with comparable anesthetic management during the maintenance of surgical state of anesthesia (n = 20) had similar power spectra in this time period (Wilcoxon Sign-Rank, p > .05), suggesting anesthetic dose rather than intrinsic susceptibility led to discontinuity. Shaded region depicts 25% to 75% confidence interval generated with bootstrap samples.

We also tested whether subjects with discontinuity received more propofol in a subset of age-matched subjects (n = 30), and found that children with discontinuity received a greater aggregate propofol bolus dose during induction (median propofol bolus: 2.1mg/kg, IQR: 1.3 to 2.4 mg.kg), compared to those without discontinuity (median propofol bolus: 0mg/kg, IQR: 0mg/kg, 97.5^th^ 1.1mg/kg); (p < .0005, Fig 2A). These results suggested that discontinuous activity was more prevalent in younger children and during periods of deeper anesthesia particularly in those that received propofol [11].

In attempt to address the possibility that certain children were inherently predisposed to manifest discontinuous activity, we compared the power spectra of children with and without discontinuity during epochs where they received comparable doses of anesthesia (i.e. during maintenance). We found that under comparable end-tidal sevoflurane concentration (median difference in etSEVO: 0.03%, IQR: -0.1 to 0.2%), age-matched subjects with and without discontinuity exhibited similar power spectra and there was no statistically significant difference between their paired spectral edges (median difference in spectral edge: 1.6 Hz, IQR: -4.6 to 4.8 Hz; n = 20, p > .05, Fig 2B). This suggested that anesthetic dose during induction, and not inherent susceptibility, contributed to discontinuous activity.

### EEG features preceding discontinuous activity

**Fig 3:**
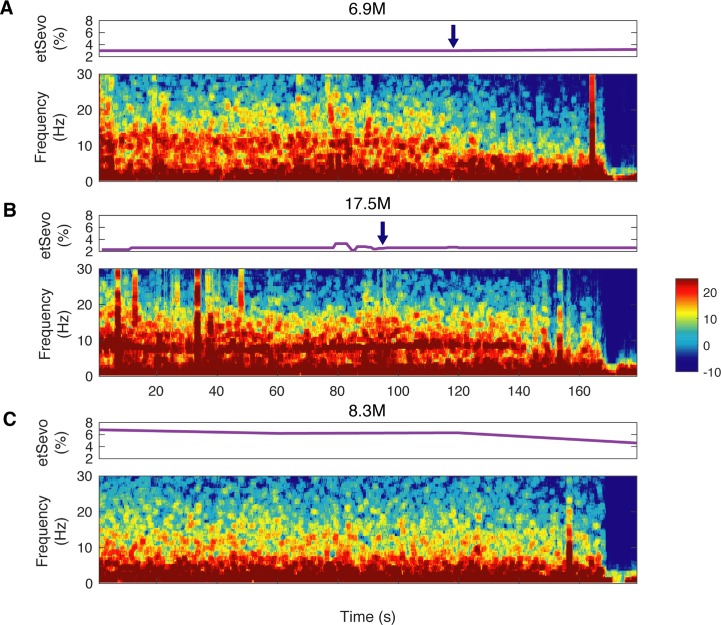
Spectrograms preceding discontinuous activity in individual subjects. Representative end tidal sevoflurane (etSevo) and spectrograms of minutes preceding first discontinuity in children at ages (A) 6 months-old, (B) 17 months-old, and (C) 8 months-old. Blue arrow indicates delivery of a propofol bolus.

Visual inspection of spectrograms in individual subjects demonstrated that consistent patterns were present before the first discontinuity (Fig 3). In particular, higher-frequency oscillations (at ~10 Hz in these three children) diminished in the tens of seconds prior to the first discontinuity. In two of these children, these reductions in power were rapid and associated with delivery of a propofol bolus (with a background of etSevo < 4%). At 6-months-old, discontinuity was evoked at etSEVO of 3% and a propofol bolus of 2.1 mg/kg (Fig 3A). At 17 months-old, discontinuity was evoked with an etSEVO of 2.6%, and a propofol bolus of 1.9mg/kg (Fig 3B). Whereas in at 8-months old, where etSEVO was given at a higher level, ranging from ~4% to 7% (median etSEVO of 6.3%), and triggered the discontinuity but with a slower reduction in power (Fig 3C).

**Fig 4:**
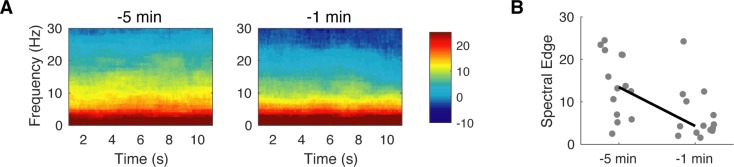
Spectral properties preceding a discontinuity event. (A) Group median spectrogram of children 4 months and older (n = 14) within five minutes and within one-minute preceding first discontinuity. (B) Spectral edge decreases within a five-minute to one-minute window preceding discontinuity (Wilcoxon Sign-Rank, p < .005). Line represents median.

To quantify this loss of higher-frequency power across subjects with discontinuity we compared the spectral edge in two 10s-time windows preceding the discontinuity event. The first in a 300s to 60s interval preceding the first discontinuity (called pseudo-baseline), and the second in the 60s preceding the first discontinuity. We have previously shown that higher-frequency power is age-dependent, with frequencies in the alpha range (8-12Hz) emerging after 3-months old, therefore we limited the analysis to include only infants older than 3-months. The median spectrograms of these time windows suggested a loss of power at higher frequencies at~ >10Hz (Fig 4A). We found that the spectral edges were lower closer to the discontinuity (median spectral edge _-300 to -60s_ discontinuity: 13.5Hz, IQR: 7 to 21.1Hz; median spectral edge _-60 to 0s_ discontinuity: 6.1Hz, IQR: 3.2 to 10.1 Hz n = 14, p<0.005); (Fig 4B). Taken together, these results suggested that spectrograms contain information that may help to predict the onset of discontinuous activity in children four months and older.

### EEG characteristics of discontinuous activity

**Fig 5:**
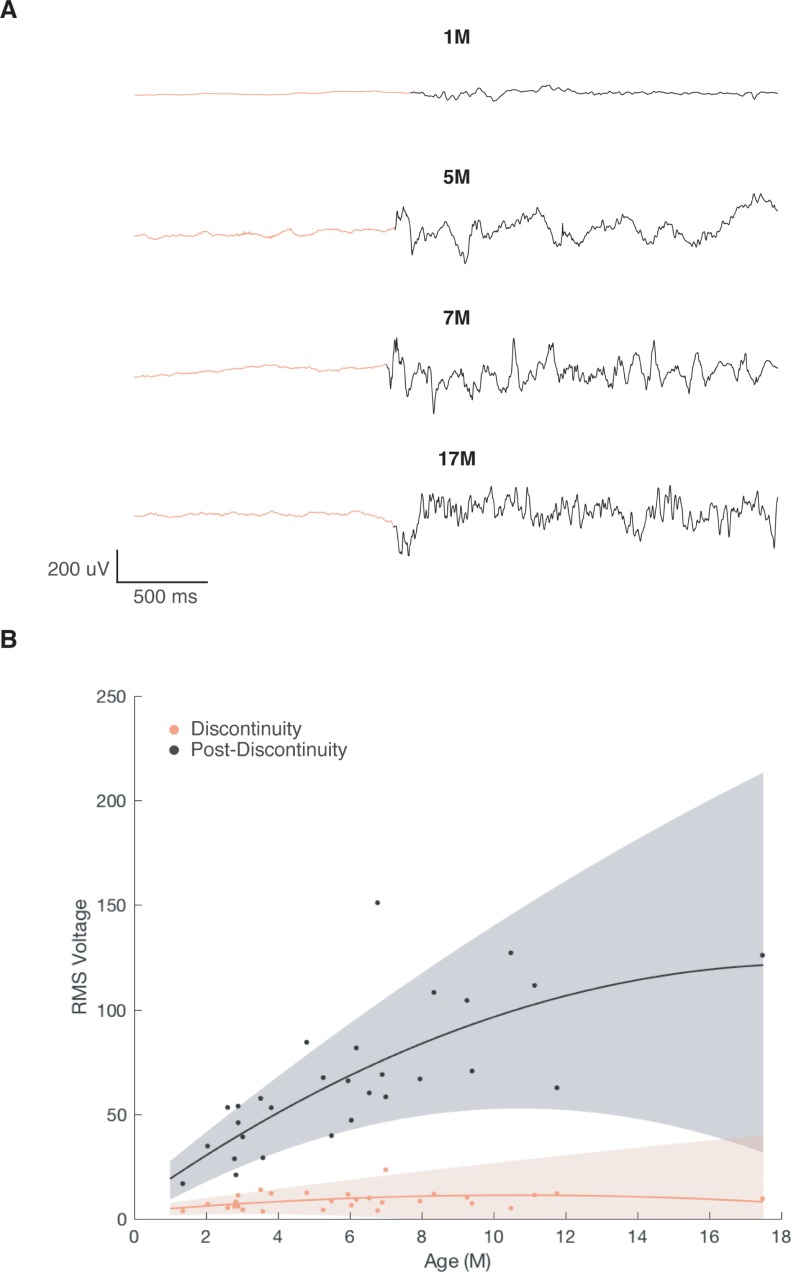
Amplitude of discontinuous activity increases with age. (A) Representative raw traces of burst-like activity following suppression in children aged 1-, 5-, 7-, and 17-months-old. (B) Root mean squared amplitude of 2s of burst-like activity following suppression increases with age (n = 29).

Previous studies have characterized various aspects of discontinuous EEGs in neonates, of which burst-suppressions are a subset, primarily focused on total time in discontinuity (or suppression) and the amplitude of post-discontinuity intervals (or bursts) [8,10,12]. We examined the amplitude of discontinuous activity. As expected, the amplitude of the post-discontinuity interval increased with age (Fig 5A), while the amplitude during discontinuity was relatively constant across ages (Fig 5B).

**Fig 6:**
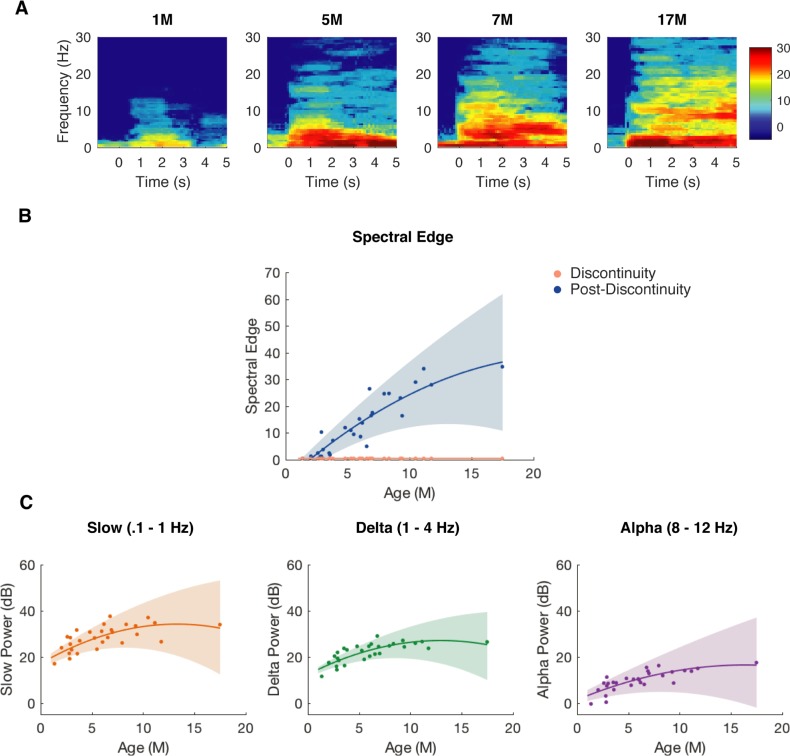
Power spectra of discontinuous activity varies with age. (A) Representative spectrograms of burst-like activity following discontinuity in children aged 1-, 5-, 7-, and 17 months-old. (B) Spectral edge of burst-like activity following suppression increases with age (n = 29) and is greater than spectral edge of suppression in children > 3-months-old. (C) Slow (.1–1 Hz), Delta (1–4 Hz), and Alpha (8–12 Hz) power all increase with age (n = 29).

We also analyzed the spectra of the discontinuous activity. Notably, individual subject spectrograms depicted in Fig 6A demonstrated that the oscillations present in the post-discontinuity interval varied with age. For example, while the post-discontinuity interval from the 1month-old subject manifested oscillations at low frequencies ~ < 4 Hz, the post-discontinuity interval from the 17 month-old subject also contained a distinct oscillation at ~ 10 Hz. We examined the spectral edge (Fig 6B) and power in canonical frequency bands (Fig 6C) and found that they all increased with age. In fact, this increased slow (.1–1 Hz), delta (1–4 Hz), and alpha (8–12 Hz) power during the post-discontinuity interval with age reflect similar developmental changes observed during the maintenance phase of anesthesia in children under sevoflurane general anesthesia [17].

**Fig 7:**
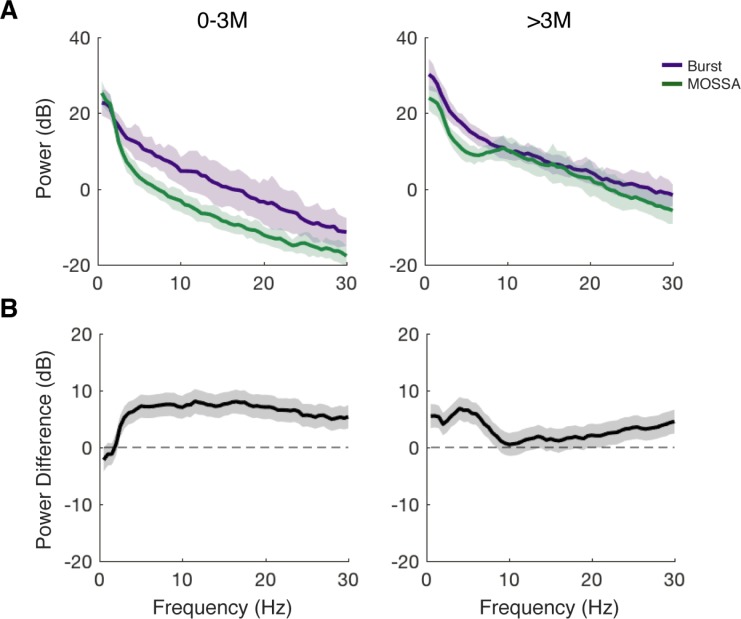
Power spectra during post-discontinuity interval and during maintenance. (A) Median power spectra of burst-like activity in children aged 0 to 3 months-old (n = 98) and >3-months-old (n = 92). Shaded region depicts 25% to 75% confidence intervals generated with bootstrap samples. (B) Differences in group median power spectra computed with bootstrap samples. Shaded region depicts 5% to 95% boundaries, and frequencies outside the shaded interval are considered significantly different. In children 0 to 3-months-old, there is negligible burst-like activity. In children >3-months-old, there is greater power in burst-like activity below 8 Hz and above 20 Hz.

We directly compared the power spectra in this post-discontinuity interval with the power spectra during maintenance and found that children 0–3 months of age exhibited an increased power greater than 2.5 Hz in the post-discontinuity interval as compared with maintenance (Fig 7). In children 4 months and greater, there was increased power below 8 Hz and above 20 Hz in the post-discontinuity interval, and no statistically significant difference between 8–20 Hz (Fig 7). These observed changes in the spectra of discontinuous activity with age suggested that discontinuous activity contains structured information that may provide insight regarding neural development state, which might impact discontinuous activity.

### Temporal changes in post-discontinuity interval

**Fig 8:**
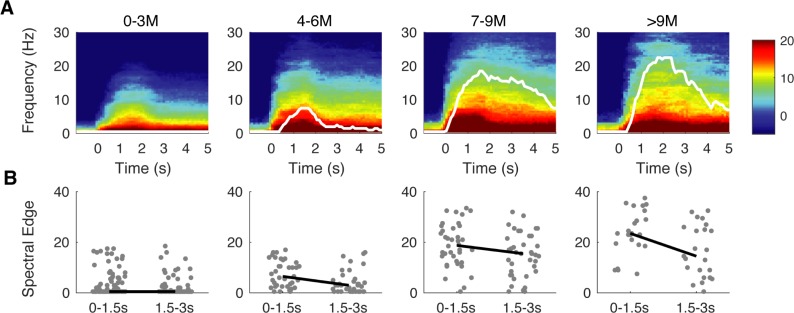
Power spectra varies over course of post discontinuity interval. (A) Group median spectrograms and spectral edges (white) of burst-like activity in children aged 0 to 3-months-old (n = 98), 4 to 6-months-old (n = 37), 7 to 9 months-old (n = 38), and >9- months-old (n = 23) show overall decrease of high frequency power in the burst-like activity. Shaded bounds of spectral edge depict standard error. (B) Spectral edge decreases from the first 1.5s (0 to 1.5s) to the next 1.5s (1.5 to 3.s) following discontinuity in all age groups as assessed by a Wilcoxon Sign-Rank test: 0 to 3 months-old (p < .000005), 4 to 6 months-old (p < .00005), 7 to 9 months-old (p < .005), >9 months-old (p < .005).

Visual inspection of the spectrograms of the post-discontinuity intervals suggested that oscillatory activity was present, and also that there was a decline in high-frequency power over the course of the post-discontinuity interval (Fig 6A). For instance, in each of the exemplar spectrograms shown, including the spectrogram of the 1month-old, the first 1.5s contained power at higher frequencies that are not present in the following 1.5s (Fig 7A). Age-grouped analysis demonstrated this decline of high-frequency power was present (Fig 8A). Spectral edge was significantly higher in the first 1.5s compared to the following 1.5s across all age groups, with the exception of the youngest infants aged 0–3 months old (Fig 8B). At 0 to 3 months-old, the group-median difference in spectral edge was 0 Hz, IQR: 0 to 2.5Hz (n = 98, p < .000005). At 4 to 6 months-old, the group-median difference in spectral edge was 2Hz, IQR: 0.4 to 6.6 Hz (n = 37, p < .00005). At 7 to 9 months-old, the group-median difference in spectral edge was 4 Hz, 95^th^ CI: 0 to 8.5 Hz (n = 38, p < .005). In the oldest subjects who were greater than 9-months-old, the group-median difference in spectral edge was 8.5 Hz, IQR: 2.6 to 12.9Hz (n = 23, p < .005).

## Discussion

Our findings provide new insight into the neurophysiology of the profoundly inactivated infant brain. We demonstrate that in children under general anesthesia, discontinuous activity is more prevalent in younger or more deeply anesthetized children and that characteristic features of this brain state vary with age.

Our findings are consistent with prior studies which indicate that both elevated levels of anesthesia and younger age are associated with discontinuous activity in children [8–10]. Profound neurodevelopmental processes occur in the first months of life which may underlie the increased susceptibility to discontinuity in the younger age groups. For instance, we found a reduced spectral edge, and power in slow, delta, and alpha frequency bands in younger children, which may reflect reduced myelination and synaptogenesis, or altered neurovascular coupling and autoregulation [5,23–26].

The impact of this discontinuous activity on neurodevelopment remains unclear. In human adults, burst suppression is associated with post-operative delirium [27–29]. However, very little is described of the association between EEG measures of discontinuity during anesthesia and short- and long-term outcomes in the human infant, especially in terms of post-operative recovery and developmental trajectories. To fill this gap, an international multicenter collaborative group announced plans to prospectively study perioperative factors associated with EEG discontinuity and post-operative outcomes in young children (NCT:03432351; estimated study completion: December 2020) [30].

Effective monitoring of depth of anesthesia is challenging in young children. Bispectral index (BIS) and entropy monitors have been proposed for use in children, but research studies have not provided reliable for infants [16]. However, juvenile animal studies examining impact of dose of anesthesia on the developing brain and associated neurologic deficits suggest that effective brain monitoring in young children may be even more important than for adults to aid appropriate anesthetic dose and to potentially reduce adverse consequences [31]. Our results suggest that the raw EEG and spectrogram may be of clinical use to assess an “over-anesthetized” brain in the absence of adequate depth of anesthesia monitors in young infants. Previous work from our group and others has characterized the distinct features of sevoflurane [14,15,20] and propofol [32,33] induced oscillations during the maintenance phase in children, and in particular the associations of specific spectral profiles with behavioral events [14,21,34]. Here we found that changes in these dynamics preceding discontinuous activity can be visualized as well. In children 4 months and older, there is a loss of higher frequency oscillations in the tens of seconds before discontinuity. In children 0–3 months of age, changes in frequency content before discontinuity could not be detected due to substantial motion artifact.

The decrease in high-frequency power over the course of the post-discontinuity interval may be similar to phenomena observed in adult burst suppression. This burst in activity can be described as an event-related synchronization, although it probably reflects activation of cortical cells which have low activity during EEG discontinuity. *Thalamic cells* that discharge synchronously rhythmic spikes to an unresponsive cortex are functionally disconnected in burst suppression (cortical afferentation) and this disrupts slow and fast rhythms in a network. Theta (5-7Hz) reflects functional preservation of corticothalamic integrity and is a sign of reactivation of neuronal networks (i.e. cortical reafferentation).

Burst suppression of the anesthetized brain has been described by neuro-metabolic models [35,36]. Along with literature regarding the occurrence of burst suppression as suggestive of harm, as noted above, burst suppression has also been a desired endpoint in the use of pentobarbital and other agents used for neuroprotection in neuro-critical care for patients with brain injuries, refractory seizures and other conditions. At the molecular level, models posit that the cycling between burst periods and quiescent periods reflects changing extracellular calcium or adenosine trisphosphate (ATP) levels, which in turn impact on membrane conductance and neurotransmitter release [35,36].

Models of adult burst suppression have predicted that there would be a deceleration of the peak frequency through the course of a burst, and that spectral activity during a burst would recapitulate the spectral activity preceding the burst suppression state [17]. While a peak frequency was difficult to discern in the children, the observed decrease in spectral edge may be related to a similar mechanism. In addition, we found that in the children 4 months of age and older, the power spectra between 8–20 Hz were similar, although there was increased power in the frequencies below 8 Hz and above 20 Hz (Fig 7). However, the children 3 months of age and younger had broadband increases in power >2.5 Hz during the post-discontinuity intervals, suggesting either that a distinct phenomenon may be present, or that they may be capable of manifesting higher-frequency activity under lighter stages of anesthesia.

Finally, we found that examining the power spectra during discontinuous activity may help to elucidate its mechanism. In particular, neuro-metabolic models of burst suppression predict that EEG properties of lighter states of anesthesia are replicated within a burst, and over the course of the burst there is a deceleration in peak frequency [17], and recent work suggests this model may also be relevant in children under anesthesia [5]. Though these exact hypotheses could not be tested in our data because of the variability in power spectra with aging, similar trends were observed in children 4 months of age and older. Notably, there was a decrease in spectral edge over the course of the post-discontinuity interval, and the frequency content of the post-discontinuity interval in children 4 months of age and older resembled lighter stages of anesthesia in the 8–20 Hz band. The spectral edge also decreased in children 0–3 months of age, though the power above 2.5 Hz in their post-discontinuity interval was higher relative to lighter anesthetic states. While our results cannot confirm or deny this model in children under anesthesia, they suggest that further exploration of these specific hypotheses is warranted, especially in children 0–3 months of age. Future computational models should examine discontinuous activity in more detail and use specific parameters incorporating knowledge of neural and vascular developmental state.

Recent investigations using multimodal approaches have begun to characterize discontinuity and burst suppression brain states in more detail. Chalia et al. found that a complex and spatially varying network of activity is present during burst suppression in infants with hypoxic ischemic encephalopathy using diffuse optical tomography, challenging the traditional notion that it is a global state [5]. These findings suggest that infant burst suppression may be similar to adult burst suppression, where electrocorticography has revealed not only a spatial, but also a distinct temporal structure in burst suppression dynamics [17]. Furthermore, these studies demonstrate that characterizing discontinuous activity, of which burst suppression is a subset, may help to shed light on the underlying neural mechanism.

EEG is a powerful tool to help understand the neurophysiology underlying anesthesia, assess anesthetic depth, and evaluate brain development at a network-level. An important limitation of this study is that it was a post-hoc exploratory analysis and was not included in the initial prospective design of the study. Additionally, initial data collection was observational, and therefore anesthetic management was not standardized across patients. In particular, administration of a propofol bolus was not consistent across subjects. Recent animal studies suggest that propofol and sevoflurane induce distinct patterns of burst suppression [37], and that this can occur in neonatal animals under anesthesia [21,38]. Likewise, distinct burst suppression patterns are also apparent in adult humans with the same agents [13,39]. Due to the confounds of age restricting our sample size, we were not able to analyze use of these agents individually. Future studies should look to evaluate local burst dynamics in infants using electrocardiography and assess the generalizability of bursts among a wider age range and patient populations.

In summary, we have demonstrated that discontinuous activity in children under general anesthesia is associated with age and anesthetic depth, and that features of discontinuous activity including spectral edge and power in canonical frequency bands vary with age. Collectively this information can help to guide future development of age-appropriate brain monitoring strategies in the pediatric operating room and intensive care unit, especially in the context of detecting changes in anesthetic depth, assessing changes in maturation, and potentially identifying biomarkers of brain stress or neurologic impairment.

## Supporting information

S1 File Source data file. Data used to generate the figures presented in this paper are openly available by downloading the accompanying Excel file.
(XLSX)

S2 File Statement of use of previously published data sets. The EEG data collected during general anesthesia have been reported elsewhere. A statement on (1) Access to previously published articles that use this data set; (2) Presentation of new data in the current article; and (3) Additional data collection for the current article, is provided in the accompanying PDF.
(PDF)
