# Supplementary material for: Electroencephalographic features of discontinuous activity in anesthetized infants and children
Source: PLoS One. 2019 Oct 3;14(10):e0223324. doi: 10.1371/journal.pone.0223324 (PMC6776336; doi:10.1371/journal.pone.0223324)
Supplement: S2 File — The EEG data collected during general anesthesia have been reported elsewhere. A statement on (1) Access to previously published articles that use this data set; (2) Presentation of new data in the current article; and (3) Additional data collection for the current article, is provided in the accompanying PDF. (PDF) [file pone.0223324.s002.pdf]

Statement on the Use of Previously Published Data Sets

A. Access to previously published articles that use this data set

The EEG data-sets collected during general anesthesia are reported elsewhere, either as full- or partial data sets depending on the research question: specifically, as a descriptive study on discontinuity during deep levels of anesthesia at 0-3 years old [1], on spectral and coherence patterns during maintenance and emergence anesthesia at 0-6 months and 0-3 years old [2,3,4], and in evaluating machine-learning algorithms to predict anesthetic concentration in neonatal animals and human infants at 0-6 months old [5]. Citations with links to the published articles are provided in Table S1.

#	Citation	Link to the published article
1	Cornelissen L, Bergin AM, Lobo K, Donado C, Soul JS, Berde CB. Electroencephalographic discontinuity during sevoflurane anesthesia in infants and children. Paediatr Anaesth. 2017 Mar;27(3):251–62.	https://doi.org/10.1111/pan.13061
2	Cornelissen L, Kim S-E, Purdon PL, Brown EN, Berde CB. Age-dependent electroencephalogram (EEG) patterns during sevoflurane general anesthesia in infants. eLife. 2015;4:e06513.	https://doi.org/10.7554/eLife.06513
3	Cornelissen L, Kim S-E, Lee JM, Purdon PL, Brown EN, Berde CB. Electroencephalographic markers of brain development during sevoflurane anesthesia in children aged 0 to 3 years old. Br J Anaesth. 2018 Jan 30;165902.	https://doi.org/10.1016/j.bja.2018.01.037
4	Cornelissen L, Donado C, Lee JM, Liang NE, Mills I, Tou A, et al. Clinical signs and electroencephalographic patterns of emergence from sevoflurane anaesthesia in children. Eur J Anaesthesiol. 2018 Jan;35(1):49–59.	https://doi.org/10.1097/EJA.0000000000000739
5	Chini M, Gretenkord S, Kostka JK, Pöpplau JA, Cornelissen L, Berde CB, et al. Neural Correlates of Anesthesia in Newborn Mice and Humans. Front Neural Circuits. 2019;13:38.	https://doi.org/10.3389/fncir.2019.00038

Table S1

B. Presentation of new data in the current article

The current article is a retrospective study with secondary analysis of previously published data describing the incidence of discontinuous EEG activity in children during sevoflurane general anesthesia (1). In this complementary paper, we present new data on the characteristics of EEG spectral and temporal properties.

C. Additional data collection for the current article

One subject previously reported on in [1] was excluded due to a technical artifact, and one additional subject was recruited and included in this analysis.
